# Some Actual Prescriptions with Their Dispensing Difficulties

**Published:** 1909-03-13

**Authors:** 


### Therapeutics and Pharmacy.

### SOME ACTUAL PRESCRIPTIONS WITH THEIR DISPENSING DIFFICULTIES.

The following are examples of some prescriptions actually sent by doctors to be made up by chemists, the latter meeting with more or less serious difficulty in dispensing the medicine as ordered:—

| | | |
|---|---|---|
| ℞ | Apomorphinæ | gr. j. |
| | Heroin Hydrochloridi | gr. j. |
| | Terebene | ʒij. |
| | Bals. Peru. | ʒj. |
| | Syrupi } Mucilaginis } | aa ad ℥ij. |

In rubbing the balsam and terebene together, prior to emulsifying with the mucilage, the terebene separated, leaving the hard, resinous portion of the balsam adherent to the mortar, so that emulsification was impossible.

A satisfactory result, however, was afterwards obtained by triturating the balsam with pulv. acaciæ ʒiij. and adding the syrup and terebene in small portions; the terebene, separating again, was poured off, and about half an ounce of water, containing the apomorphine and heroin hydrochloride, was added to the thin emulsion. Finally, adding the separated terebene again, a good emulsion was obtained.

| | | |
|---|---|---|
| ℞ | Spt. Ammon. Aromat. | ℥ss. |
| | Potass. Bromid. | ʒiij. |
| | Glycerini | ℥j. |
| | Aq. Chloroformi | ad ℥ij. |

There being more potassium bromide than can be taken into solution, the chemist powdered the salt and labelled the mixture "To be shaken," although he would have preferred to replace half of the glycerin with water, thus ensuring complete solution of the salt.

| | | |
|---|---|---|
| ℞ | Potass. Chlor. | ʒj. |
| | Ac. Sulphuros. | ʒiij. |
| | Glycerini | ℥ss. |
| | Aq. | ad ℥vj. |

This prescription was referred to the prescriber, attention being drawn to the incompatibility.

| | | |
|---|---|---|
| ℞ | Lithii Salicyl. } Sodii Hyposulph. } Sodii Sulphat. exsiccati } Potass. Cit. } | aa ʒiv. |
| | Potass. Bicarb. | ℥viij. |

No directions were given as to the form it was desired this prescription should take. The chemist finally powdered the hyposulphite, mixed it with the other powders, and granulated the mixture over a water-bath.

| | | |
|---|---|---|
| ℞ | Acid. Hydrocyan. dil. | ʒj. |
| | Liq. Bismuthi | ʒvj. |
| | Tinct. Capsici | ʒss. |
| | Glycerin. Pepsin | ad ℥iij. |
| | Ft. mist. | |

This, if made with B.P. preparations, always yields a precipitate because the hydrochloric acid of the glycerinated pepsin combines with the ammonium-bismuth-citrate of the liquor bismuthi, insoluble bismuth citrate being deposited. Commercial liquor bismuthi is often unduly alkaline, and with it no such precipitate follows the mixing; the commercial product contains enough free ammonia to neutralise the hydrochloric acid of the pepsin. With neutral liquor (B.P.) it is necessary to add a trace of ammonia, and then the precipitated bismuth citrate redissolves at once.

| | | |
|---|---|---|
| ℞ | Ferri Carb. Sacch. | gr. iij. |
| | Ferri Arseniat. | gr. $\frac{1}{8}$ |
| | Ext. Gentian. | gr. $\frac{1}{2}$ |
| | Ft. pil. | |

This pill splits after finishing. Why should this be so? Splitting of pills is usually caused by liberation of gas, often met with in iron pills, grey-powder pills, and others which have been massed with acid excipients. In this pill the extract has been acid and it has set free a little $CO_2$ from the saccharated carbonate of iron.

| | | |
|---|---|---|
| ℞ | Bism. Subnit. | ʒij. |
| | Acid. Carbolic. | gr.x. |
| | Paraff. Moll. | ad ℥ij. |
| | Ft. ung. | |

This ointment in some towns becomes black on keeping. The reason for this probably is that the water in these places contains traces of iron; a little ferruginous water mixed with the above ingredients would lead to the formation of dark carbonate of iron. If the phenol be dissolved in the liquefied paraffin, and the bismuth subnitrate be then added dry, the ointment does not become discoloured on keeping.

| | | |
|---|---|---|
| ℞ | Potass. Bromid. | ʒij. |
| | Tinct. Gelsemii | ʒss. |
| | Aq. Camph. | ad ℥iij. |
| | Ft. mist. | |

The above is a common headache mixture. It is clear when first dispensed, but on standing a dark deposit forms. What is it, and how should the prescription be altered so as to avoid it?

The deposit most likely consists of resin and camphor salted out by the potassium bromide. Probably the trouble would be overcome by replacing the camphor water by chloroform water.

| | | |
|---|---|---|
| ℞ | Bismuth, Subnit. | gr. v. |
| | Potass. Bichromat. | gr. $\frac{1}{6}$ |
| | Ft. pil. | |

What excipient is likely to be the best for this pill? Soft paraffin, or paraffin ointment, made as firm as may be necessary with kaolin, is the best. Potassium bichromate, potassium permanganate, silver nitrate, and similar easily reduced substances cannot be dispensed with any of the commoner excipients; they may be made up with anhydrous wool fat if the pills are to be used quickly; on keeping, slow reduction of potassium bichromate occurs if it is so massed, the change taking place from without inwards as the anhydrous fat gradually absorbs moisture.

---

Tinctura ferri perchloridi loses its colour on keeping, whereas liquor ferri perchloridi does not. This is, of course, because the alcohol of the tincture is a reducing substance and the ferric chloride is an oxidiser. Exposure to light induces or accelerates action between the two, the ferric iron becoming ferrous, and the chlorine oxidising or combining with the alcohol.

*(To be concluded.)*

